# Tumor-Derived CXCL1 Promotes Lung Cancer Growth via Recruitment of Tumor-Associated Neutrophils

**DOI:** 10.1155/2016/6530410

**Published:** 2016-06-29

**Authors:** Ming Yuan, Ha Zhu, Junfang Xu, Yuanyuan Zheng, Xuetao Cao, Qiuyan Liu

**Affiliations:** ^1^National Key Laboratory of Medical Immunology & Institute of Immunology, Second Military Medical University, Shanghai 200433, China; ^2^Institute of Immunology, Zhejiang University School of Medicine, Hangzhou 310058, China

## Abstract

Neutrophils have a traditional role in inflammatory process and act as the first line of defense against infections. Although their contribution to tumorigenesis and progression is still controversial, accumulating evidence recently has demonstrated that tumor-associated neutrophils (TANs) play a key role in multiple aspects of cancer biology. Here, we detected that chemokine CXCL1 was dramatically elevated in serum from 3LL tumor-bearing mice.* In vitro*, 3LL cells constitutively expressed and secreted higher level of CXCL1. Furthermore, knocking down CXCL1 expression in 3LL cells significantly hindered tumor growth by inhibiting recruitment of neutrophils from peripheral blood into tumor tissues. Additionally, tumor-infiltrated neutrophils expressed higher levels of MPO and Fas/FasL, which may be involved in TAN-mediated inhibition of CD4^+^ and CD8^+^ T cells. These results demonstrate that tumor-derived CXCL1 contributes to TANs infiltration in lung cancer which promotes tumor growth.

## 1. Introduction

Lung cancer is the most commonly diagnosed malignancy with approximately 224,000 new cases in 2014 in western world [1]. Although great advances have been made in the early detection and therapeutic approaches, the morbidity of lung cancer is still raising and leading to the first cause of death in cancer [1]. Chronic inflammation is strongly linked to lung cancer initiation and progression. In lung cancer patients or experimental animal models, the composition and phenotype of neutrophils/granulocytic cells in blood are dramatically altered [2, 3]. Multiple evidence shows that neutrophils are presented in the tumor sites or paratumor tissue in various types of tumors, and the density of neutrophils is an independent factor for predicting the prognosis of cancer patients [4]. Tumor-derived chemokines and cytokines recruit myeloid cells into tumor microenvironment and educate them into protumor phenotype [5]. However, the mechanisms that are involved in the recruitment of neutrophils to the tumor microenvironment are far from clear. In hepatocellular carcinoma (HCC), tumor-derived chemokine CXCL5 mediate the recruitment of neutrophils, and the density of TANs is associated with poor prognosis of cancer patients [6]. The chemokine receptor CXCR2 is expressed in neutrophils/granulocytic cells. When CXCR2 interacts with its ligand, neutrophils are recruited to the inflamed site [7]. Additionally, Lewis lung carcinoma cells-derived oxysterol plays a key role in the recruitment of CXCR2^+^ tumor-promoting neutrophils into tumor tissues [8]. Tumor-expressed chemokines CXCL8 and CXCL6 are also involved in the neutrophils infiltration [9].

Chemokine (C-X-C motif) ligand 1 CXCL1 (also referred to as GRO-1) binds to CXCR2, which is highly expressed on the surface of neutrophils [10]. In both infection and cancer microenvironment, CXCL1 is elevated by various stress-inducing factors, including PGE2 [11]. Elevated levels of CXCL1 and CXCR2 positively correlate with the poorer prognosis of cancer patients [12, 13]. It has been reported that CXCL1/CXCR2 density is strongly associated with the number of neutrophils in the tumor microenvironment and can be an independent factor for predicting the prognosis of patients with hepatocellular carcinomas [14]. In animal studies, CXCL1 receptor CXCR2 deficiency prevents from tumorigenesis in colitis-associated cancer [15] and colorectal cancers [16] by inhibiting myeloid-derived suppressive cells (MDSCs) infiltration. Furthermore, CXCL1-mediated myeloid cells infiltration is associated with therapeutic response in breast cancer [17]. Recently, it has been reported that stably silencing of CXCL1 can inhibit tumor growth in HCC [18], and knocking down of CXCL1 expression can inhibit tumor growth in colorectal liver metastasis [19]. Additionally, autocrine and paracrine of CXCL1 can also promote tumor invasion and metastasis [17, 20, 21].

In this study, we found that the level of CXCL1 in serum was significantly upregulated in 3LL lung cancer bearing mice. Knocking down CXCL1 expression in 3LL cells significantly inhibited neutrophils infiltration, resulting in reducing tumor growth* in vivo*. Tumor-infiltrated neutrophils in tumor tissues expressed higher levels of MPO and Fas/FasL, which may be involved in TAN-mediated inhibition of CD4^+^ and CD8^+^ T cells. In conclusion, tumor-derived CXCL1 contributes to neutrophils infiltration in lung cancer which promotes tumor growth.

## 2. Materials and Methods

### 2.1. Mice and Cell Lines

C57BL/6J mice (6–8 weeks) were obtained from Joint Ventures Sipper BK Experimental Animal Company (Shanghai, China). All experimental manipulations were undertaken in accordance with the National Institutes of Health Guide for the Care and Use of Laboratory Animals, with the approval of the Scientific Investigation Board of the Second Military Medical University, Shanghai, China. A Lewis lung carcinoma sub-line 3LL was maintained as described previously [22]. A mouse epithelial cell line MLE and a human non-small lung carcinoma cell line A549 were purchased from ATCC.

### 2.2. CXCL1 Knockdown

3LL cells (2 × 10^5^/well) were plated into 6-well plates and transfected with negative control (NC) or CXCL1-specific shRNA plasmids (design by Gene Pharma, China) using jetPEI (Polyplus-transfection, France) as described previously [23]. Stably silenced transfected cell clones were selected in 800 *μ*g/mL G418 for 14 days; GFP-expressing cells were selected by FACS sorting system and analyzed by real-time PCR and ELISA for CXCL1 expression and secretion.

### 2.3. Flow Cytometric Analysis

For analysis of tumor-infiltrating cells, tumor-infiltrating lymphocytes (TILs) were isolated from transplanted tumors and suspended into single cells. For analysis of the compartment of immune cells in the peripheral blood, blood was collected in a tube with heparin and incubated with Tris-NH_4_Cl to remove erythrocytes. Cells were stained with monoclonal anti-mouse antibodies as follows: CD45-BV510, CD3*ε*-PE-Cy7, CD8*α*-PE, CD4-PE-Cy5, CXCR2-PerpCy5.5, CD11b-APC, Ly6G-PE, Ly6C-FITC, Fas-PE, FasL-PE, CD11b-PerCP-Cy5.5, CD8a-FITC, 7-AAD, and Annexin-V-FITC. Flow cytometry analysis was carried out using a BD FACS (BD Biosciences).

### 2.4. ELISA

Mouse and human CXCL1 ELISA kit (R&D systems) were used to determine the concentrations of CXCL1 in cell culture supernatant or serum from tumor-bearing mice according to the manufacturer's instructions.

### 2.5. qRT-PCR

Total mRNA of 3LL cells was extracted with Trizol reagent (Invitrogen) and then subjected to reverse transcribed by using M-MLV reverse transcribed kit (Takara). cDNA was amplified by ABI7300 Detection System (Applied Biosystems) using SYBR Green PCR kit (Takara). Levels of CXCL1 were normalized by the levels of *β*-actin in each individual sample. Specific primers were as follows:

 m*β*-actin: forward (5′-AGTGTGACGTTGACATCCGT-3′),
 m*β*-actin: reverse (5′-GCAGCTCAGTAACAGTCCGC-3′),
 mCXCL1: forward (5′-GTCATAGCCACACTCAAGAA-3′),
 mCXCL1: reverse (5′-AGACAGGTGCCATCAGAG-3′).

### 2.6. Chemotaxis Assay

Cell migration was estimated by using a pore size of 3 *μ*m transwell chambers Matrigel (BD Biosciences, Bedford, MA, USA). 2 × 10^6^ cells isolated from spleen and peripheral blood in 200 *μ*L of serum free medium were added into the upper chamber, and 600 *μ*L of cell culture supernatant from 3LL/NC, 3LL/shCXCL1, or medium control was added into bottom chamber. After incubating for 4 h, the cells phenotype on the bottom was analyzed by flow cytometric analysis.

### 2.7. Tumor-Bearing Model

5 × 10^5^ 3LL/shCXCL1 cells or 3LL/NC cells were injected subcutaneously in the abdomen of C57BJ/6L mice, tumor volume (V) was measured twice a week and calculated by using the formula 0.5 × [Length × Width^2^].

### 2.8. Immunofluorescence (IF)

CD11b^+^Ly6G^+^ neutrophils in tumor tissue sections from tumor-bearing mice were analyzed by immunofluorescence staining. Ly6G antibody was used at the dilution of 1 : 50. The fluorescent-labeled second antibodies were used at the dilution of 1 : 100. Digital imaging was carried out using the software LASV4.5 (Leica DM 2000).

### 2.9. Immunohistochemistry (IHC)

Five-micrometer thick sections were cut from formalin-fixed and paraffin-embedded tissue specimens and put onto slides; sections were deparaffinized through alcohol gradients and rehydrated to water. Antigens were retrieved by using Citrate pH 6.0 buffer in thermostatic bath at 100°C for 5 minutes. Tissue sections were incubated with primary anti-mouse Ly6G (Biolegend) at the dilution of 1 : 50 or anti-mouse MPO (Abcam) at the dilution of 1 : 200. Rabbit polyclonal IgG (DAKO) was used as negative control instead of primary antibody. Digital imaging was examined using the software LAS V4.5 (Leica DM 2000).

### 2.10. Coculture System

TANs were isolated from tumor tissues derived from 3LL/NC tumor-bearing mice; CD4^+^ T cells were isolated from the spleens of naïve mice. Cell sorting were carried out by using MACS system (Miltenyi Biotech). Splenic CD4^+^ T cells (5 × 10^4^/well) with or without neutrophils (5 × 10^4^/well) were cocultured in precoated CD3 (2 *μ*g/mL) 24-well plates with soluble CD28 (2 *μ*g/mL) and murine recombinant IL-2 (1 ng/mL) for 3 days. CD4^+^ T cells number was counted by flow cytometry.

### 2.11. Statistic Analysis

All the statistics were analyzed with the assistance of Graphpad Prism 5.0. The comparisons between two groups were analyzed by unpaired Student's *t*-test.

## 3. Results

### 3.1. Increased Level of CXCL1 in Serum Is Mainly Derived from 3LL Cancer Cells* In Vivo*

**Figure 1:**
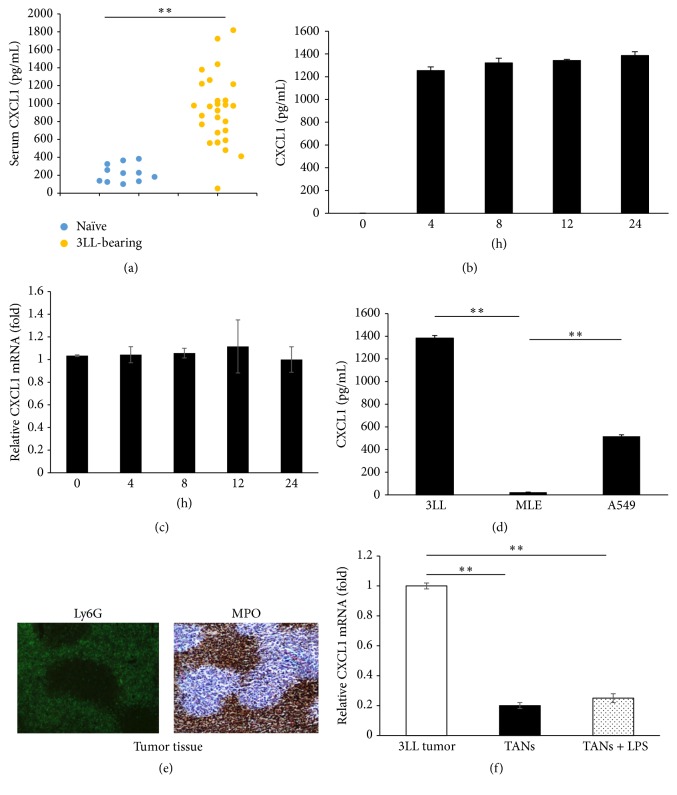
Expression and secretion of CXCL1* in vitro *and* in vivo*. (a) Levels of CXCL1 in the serum of naive mice (*n* = 11) or 3LL tumor-bearing mice (*n* = 27) were measured by ELISA at 14 days after tumor inoculation. Each dot represents an individual mouse. (b) CXCL1 protein levels in the 3LL culture supernatants were determined by ELISA. (c) mRNA expression of CXCL1 in 3LL cells was measured by quantitative RT-PCR. (d) ELISA analysis of CXCL1 production in Lewis lung carcinoma cell line 3LL, mouse epithelial cell line MLE, and human non-small lung carcinoma cell line A549. (e) Ly6G^+^MPO^+^ neutrophils in 3LL tumor tissues were determined by IF and IHC. (f) Quantitative RT-PCR analysis of CXCL1 expression in 3LL tumor cells and TANs (sorted from 3LL tumor tissues) stimulated with or without LPS (100 ng/mL) for 6 hours. ^*∗∗*^ *p* < 0.01; data are shown as mean ± SD.

**Figure 2:**
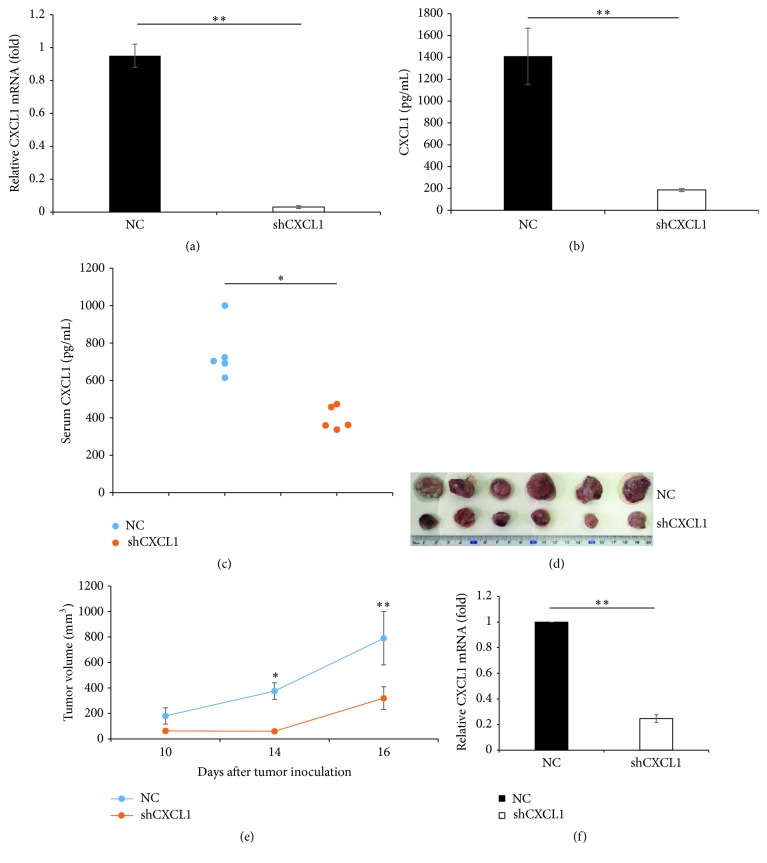
Ablation of CXCL1 in 3LL cells limits tumor growth* in vivo*. (a) 3LL cells were stable transfected with negative control shRNA (NC) or specific CXCL1 shRNA (shCXCL1); CXCL1 mRNA expression was measured by quantitative RT-PCR. (b) Protein levels of CXCL1 in the 3LL/NC and 3LL/shCXCL1 supernatants were measured by ELISA. (c) Serum levels of CXCL1 from 3LL/NC or 3LL/shCXCL1 bearing mice were measured by ELISA. Each dot represents an individual mouse. (d) Primary tumors excised from 3LL/NC bearing mice and 3LL/shCXCL1 bearing mice at day 16 after tumor inoculation. (e) Growth of NC and shCXCL1 tumors was monitored for 16 days after cell inoculations. (f) CXCL1 mRNA expression in tumor tissues derived from 3LL/NC or 3LL/shCXCL1 tumor-bearing mice was examined by quantitative RT-PCR. ^*∗*^ *p* < 0.05, ^*∗∗*^ *p* < 0.01. Results are shown as mean ± SD.

Compared to naïve mice, serum levels of CXCL1 in 3LL lung cancer bearing mice were significantly increased (Figure 1(a)). To examine the source of serum CXCL1 in 3LL tumor-bearing mice, we first examined the expression and secretion of CXCL1 in 3LL cells and found that 3LL cells constitutively secreted and expressed high level of CXCL1 (Figures 1(b) and 1(c)). In addition, we found the human non-small lung carcinoma cell A549 also secreted higher levels of CXCL1 (Figure 1(d)). However, the mouse epithelial cell line MLE did not secrete CXCL1 (Figure 1(d)), suggesting lung cancer cells could express and secrete higher levels of CXCL1. It has been reported that neutrophils are also able to express and produce significant amounts of CXCL1 when activated [24, 25]. Larger amounts of Ly6G^+^MPO^+^ neutrophils were found in 3LL tumor tissues (Figure 1(e)). Then, we analyzed CXCL1 expression in TANs and 3LL tumor. The results showed that the expression of CXCL1 in 3LL tumor was higher than that in TANs or LPS-stimulated TANs (~9-fold) (Figure 1(f)). Furthermore, we knocked down CXCL1 expression in 3LL cells using CXCL1 targeted shRNA construct. The results showed that compared to negative control (NC), CXCL1 mRNA expression and secretion in CXCL1-silencing 3LL cells (shCXCL1) were significantly decreased (Figures 2(a) and 2(b)). Serum levels of CXCL1 were markedly decreased in the mice inoculated with shCXCL1 cells (Figure 2(c)). Taken together, these results demonstrate that 3LL-derived CXCL1 is the main source that contributes to the increased serum level of CXCL1* in vivo*.

### 3.2. Knockdown of CXCL1 Expression in 3LL Cells Inhibits Tumor Growth* In Vivo*

Then, to investigate the roles of CXCL1 in lung cancer growth* in vivo*, 3LL transfected cells (NC or shCXCL1) were subcutaneously inoculated into C57BL/6J mice. The results showed that 3LL negative control cells (NC) grew aggressively* in vivo*, while knockdown CXCL1 expression in 3LL cells (shCXCL1) significantly attenuated tumor growth (Figures 2(d) and 2(e)). Furthermore, quantitative PCR analysis showed that CXCL1 expression in 3LL/shCXCL1 tumor tissues was significantly lower than that in 3LL/NC tumor tissues (Figure 2(f)). These results indicate that tumor-derived CXCL1 contributes to lung cancer growth* in vivo*.

### 3.3. Tumor-Derived CXCL1 Mediates TANs Infiltration

**Figure 3:**
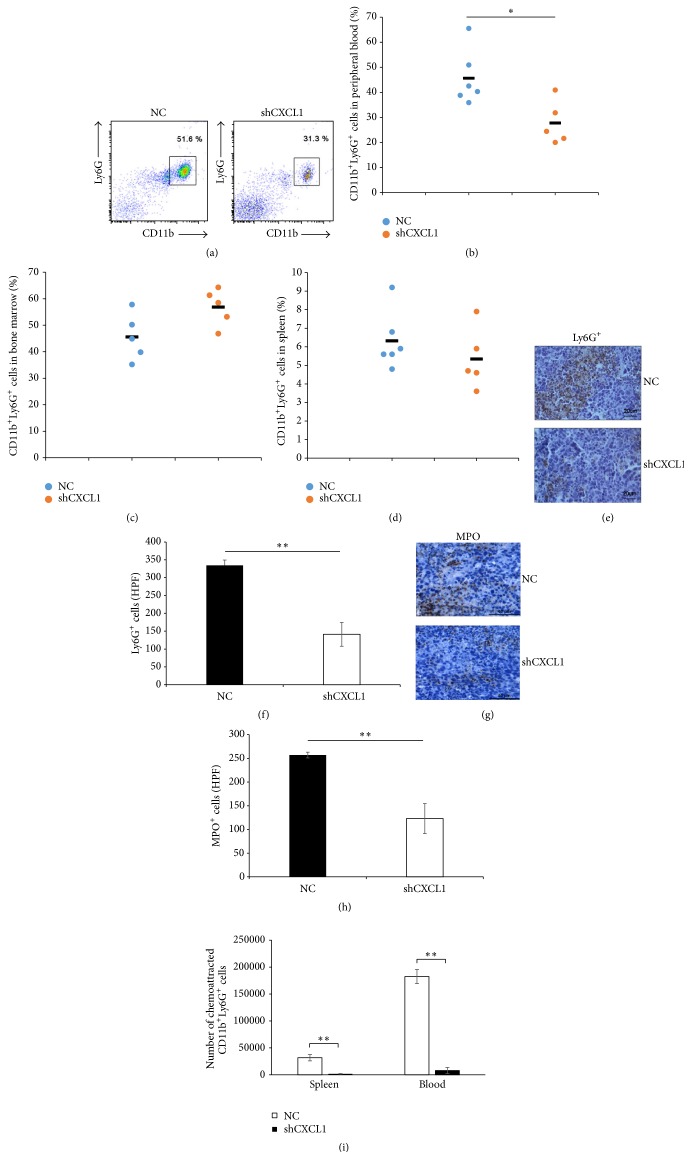
Knockdown of CXCL1 expression in 3LL cells decreases the number of neutrophils* in vivo*. 5 × 10^5^ 3LL/NC or 3LL/shCXCL1 cells were inoculated subcutaneously (s.c.) into C57BL/6J mice. At day 16, mice were scarified. The CD11b^+^Ly6G^+^ neutrophils in peripheral blood were detected as described in the materials and methods. Representative flow cytometric graph (a) and percentage of CD11b^+^Ly6G^+^ neutrophils in peripheral blood (b) were analyzed. Each dot plot represents an individual mice; horizontal lines are shown as mean of each group. (c) Flow cytometric analysis of the frequency of CD11b^+^Ly6G^+^ neutrophils in bone marrow from mice inoculated with 3LL/NC or 3LL/shCXCL1. (d) Flow cytometric analysis of the frequency of CD11b^+^Ly6G^+^ neutrophils in spleen from mice inoculated with 3LL/NC or 3LL/shCXCL1 tumor cells. (e, f, g, and h) IHC staining of Ly6G^+^ or MPO^+^ cells in the tumor tissues from mice inoculated with 3LL/NC or 3LL/shCXCL1 tumor cells. IHC staining quantification of Ly6G^+^ (f) or MPO^+^ (h) cells (per high-power field, HPF). Scale bars, 20 *μ*m or 40 *μ*m. (i) Neutrophils were isolated from spleen or peripheral blood derived from tumor-bearing mice and the chemoattracted CD11b^+^Ly6G^+^ neutrophils were analyzed towards the condition supernatant from NC and shCXCL1 tumor cells by* in vitro* transwell assay. ^*∗*^ *p* < 0.05, ^*∗∗*^ *p* < 0.01.

CXCL1 is an important chemokine that contributes to recruitment of CXCR2 expressing myeloid cells and neutrophils [7]. Myeloid cells in mice consist of CD11b^+^Ly6C^+^ monocytic cells and CD11b^+^Ly6G^+^ neutrophils/granulocytic cells [26]. Flow cytometric analysis showed that the frequency of CD11b^+^Ly6G^+^ neutrophils in the circulation of mice inoculated 3LL/shCXCL1 (44.6% ± 4.45%) was significantly decreased compared to mice inoculated with 3LL/NC (57.7% ± 1.8%) (Figures 3(a) and 3(b)). However, CD11b^+^Ly6G^+^ neutrophils counts in bone marrow were similar (NC versus shCXCL1; 41.88% ± 3.7% versus 53% ± 3.82%) (Figure 3(c)). In addition, CD11b^+^Ly6G^+^ neutrophils counts in spleen of mice inoculated with 3LL/NC tumor cells did not profoundly change compared to mice inoculated with 3LL/shCXCL1 tumor cells (NC versus shCXCL1; 6.31% ± 1.55% versus 5.34% ± 1.65%) (Figure 3(d)). IHC staining of Ly6G showed that the number of Ly6G^+^ neutrophils in tumor tissues from mice inoculated with 3LL/NC was higher than that in 3LL/shCXCL1 bearing mice (Figures 3(e) and 3(f)). As myeloperoxidase (MPO) is signature marker of neutrophils existing in the granules of neutrophils [27], the result showed that there were more MPO^+^ TANs from mice inoculated with 3LL/NC than that from mice inoculated with 3LL/shCXCL1 (Figures 3(g) and 3(h)). Furthermore, using a transwell system, the accumulation of CD11b^+^Ly6G^+^ neutrophilsby CXCL1* in vitro* was detected. The results showed that knockdown of CXCL1 expression in 3LL cells markedly reduced the number of chemoattracted neutrophils (chemoattracted neutrophils counts isolated from spleen: 3LL/NC medium versus 3LL/shCXCL1 medium 31784 ± 5904 versus 1804 ± 582) (chemoattracted neutrophils counts isolated from blood: 3LL/NC medium versus 3LL/shCXCL1 medium 182502 ± 13119 versus 7992 ± 5763) (Figure 3(i)). These results demonstrate that tumor-derived CXCL1 promotes neutrophils recruitment* in vitro *and* in vivo*.

### 3.4. Tumor-Infiltrated Neutrophils Inhibit T Cell-Mediated Anti-Tumor Function* In Vitro* and* In Vivo*

**Figure 4:**
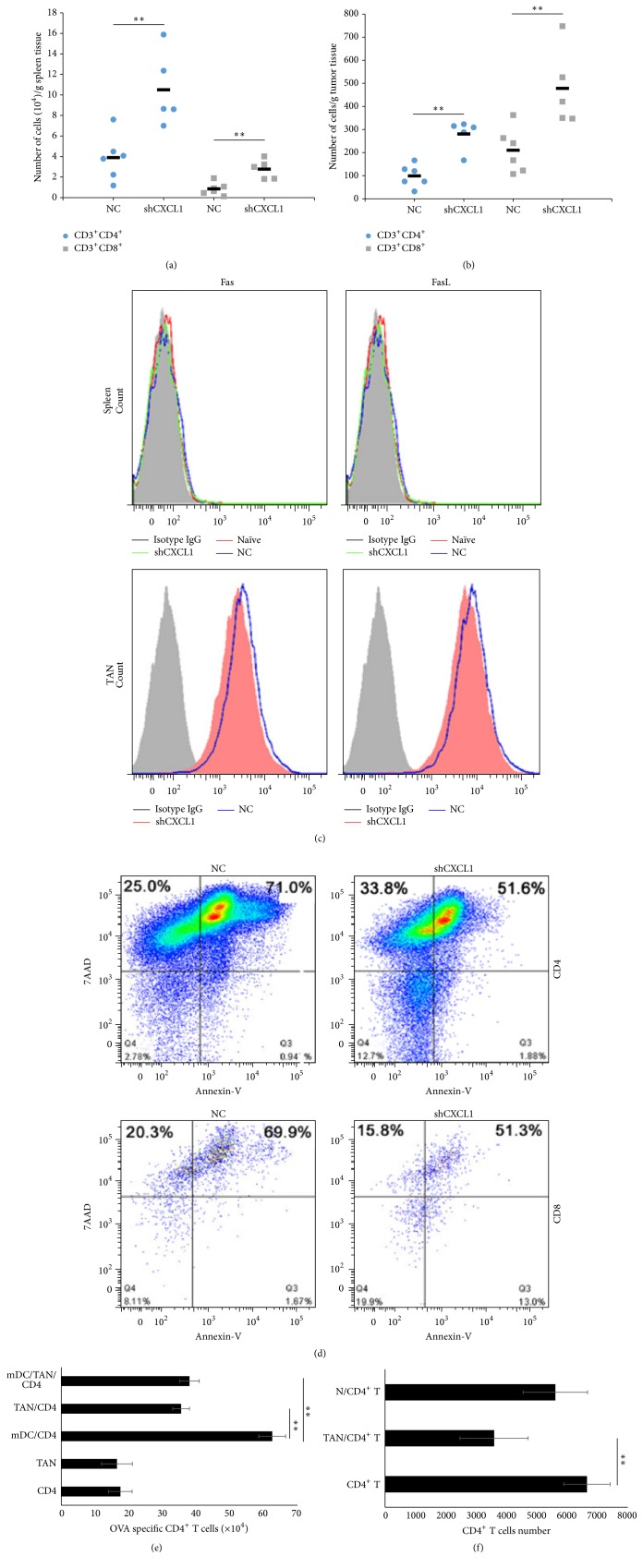
Tumor-infiltrating neutrophils in tumor inhibit T cell proliferation. Number of CD3^+^CD4^+^ and CD3^+^CD8^+^ T cells in the spleen (a) and tumor tissues (b) at day 14 after tumor inoculation were analyzed by flow cytometric. (c) Fas/FasL expression on splenic CD11b^+^Ly6G^+^ neutrophils or TANs from 3LL/NC bearing mice, 3LL/shCXCL1 bearing, or naïve mice were analyzed by cytometric analysis. (d) CD4^+^ T or CD8^+^ T cells derived from tumor tissues were stained with 7AAD and Annexin-V, flow cytometric analysis of apoptotic/necrotic CD4^+^ T, and CD8^+^ T cells. (e) Splenic CD4^+^ T plus conventional dendritic cells together with or without CD11b^+^Ly6G^+^ neutrophils were cocultured for 3 days with 10 *μ*g/mL OVA; CD4^+^ T cells were counted by flow cytometry. (f) Splenic CD4^+^ T and TANs were cocultured for 3 days with anti-CD3 and anti-CD28 and IL-2, CD4^+^ T cells numbers were counted by flow cytometry. N (splenic neutrophils); TAN (tumor-associated neutrophils). ^*∗∗*^ *p* < 0.01. Data are presented as means ± SD.

Previous study reported that TANs exhibited protumor phenotype by suppressing activation of CD8^+^ T cells in lung cancer [28]. So, we detected the number of effector T cells in spleen and tumor tissues derived from tumor-bearing mice. The results showed increased number of both CD3^+^CD4^+^ and CD3^+^CD8^+^ T cells in spleen and tumor tissues in 3LL/shCXCL1 group compared to 3LL/NC control group (Figures 4(a) and 4(b)), suggesting that the higher density of neutrophils was paralleled with the decreased T cells number in lung cancer. It has been reported that Fas/FasL expression by neutrophils can directly regulate CD8^+^ T cells infiltration [29]. We analyzed the expression of Fas/FasL in splenic neutrophils or TANs. As shown in Figure 4(c), splenic neutrophils expressed lower levels of Fas/FasL; however, tumor infiltrated neutrophils derived from both 3LL/NC and 3LL/shCXCL1 bearing mice expressed higher levels of Fas/FasL. Flow cytometric analysis demonstrated that the number of apoptotic/necrotic CD4^+^ T cells and CD8^+^ T was lower in 3LL/shCXCL1 group compared to 3LL/NC group (Figure 4(c)). Furthermore, to determine the effect of TANs on proliferation of T cells, we isolated TANs in tumor tissues from 3LL tumor-bearing mice. TANs significantly inhibited mature dendritic cells- (mDC-) mediated CD4^+^ T proliferation* in vitro *(Figure 4(e)). Consistently, TANs suppressed CD4^+^ T proliferation primed by anti-CD3 and CD28 (N/T cells, TANs/T cells versus T cells; 5612.3 ± 1487.4, 3592 ± 1122.5, versus 6652.7 ± 764.9) (Figure 4(f)). These results suggest that tumor infiltrated neutrophils could inhibit the proliferation and induce apoptosis/necrosis of T cells.

## 4. Discussion

Proinflammatory and chemokine factors play a prominent role in lymphocyte homing and migration [30–32]. Accumulating evidence demonstrated that tumor-derived chemokines modified the compartment of myeloid cells in the tumor microenvironment. CCL2 induced infiltration of regulatory dendritic cells and regulatory T cells in the tumor microenvironment [33], CXCL5/CXCR2 axis mediates the accumulation of CXCR2^+^ MDSCs [34], CXCR2^−/−^ mice were resistance to colitis-associated cancer formation by inhibiting MDSCs accumulation in the mucosa [15], and silence of CXCL1/2 expression in breast cancer decreased the infiltration of myeloid cells in the tumor microenvironment [17]. However, there is little evidence directly elucidating the function of tumor-derived CXCL1 on the migration of neutrophils/granulocytic cells. In this study, we demonstrated that 3LL tumor-derived chemokine CXCL1 regulated the composition of immune cells in tumor microenvironment. Knockdown of CXCL1 in 3LL cells resulted in decreased number of TANs, paralleled by increased number of CD4^+^ T and CD8^+^ T cells. So, we demonstrated that tumor-derived CXCL1 increased the number of Ly6G^+^ neutrophils in the peripheral blood and tumor tissues. Silencing CXCL1 expression and secretion in 3LL cells markedly reduced the number of Ly6G^+^ neutrophils. Accordingly, CXCL1 deficiency in tumor significantly inhibited tumor growth* in vivo*. It has been well known that the increased number of neutrophils in peripheral blood is due to increased egress of bone marrow or prolonged life span [35]. So, we further examined the number and proportion of neutrophils in bone marrow. The results showed that there was no statistical significant difference of density of neutrophils in bone marrow between mice inoculated with 3LL/NC and inoculated with 3LL/shCXCL1; however, there is approximately 1.3-fold increase in neutrophil counts in the bone marrow of 3LL/shCXCL1 tumor-bearing mice, suggesting that CXCL1 maybe determine the efficiency of neutrophil egress from bone marrow. Although more evidence has identified the potential role of neutrophils in cancer progression. The roles of neutrophils in tumor microenvironment have not been well demonstrated [36]. Matrix metalloproteinases (MMPs), such as MMP9, mainly produced by neutrophils, have the ability to degrade the extracellular matrix (ECM), which contribute to carcinogenesis and tumor progression [37, 38]. However, neutrophils-derived MMP-8 was regarded as playing a protective role in tumor progression [39]. In a spontaneous metastasis model, deletion of neutrophils in tumor microenvironment decreased tumor angiogenesis and intravasation [40]. In addition, TANs promoted tumor invasion by releasing of ROS [41], growth factors such as hepatocyte growth factor (HGF) [42], and cytokines like oncostatin M [43]. Orchestrating antitumor immunity in the tumor environment is another important function of TANs. It was proven that neutrophils inhibited T cell proliferation by releasing intracellular arginase I in non-small lung cancer [44]. In mouse lung tumors, neutrophils depletion leaded to more activated CD8^+^ T cells intratumorally [28]. Multiple studies have demonstrated that TANs and lymphocytes ratio in tumor microenvironment could be a prognostic predictor in patients with various types of cancer, including lung carcinoma [45, 46] and esophageal carcinoma [47]. Our results demonstrated that the increased number of neutrophils was associated with decreased T cells number in tumor microenvironment. CD11b^+^Ly6G^+^ neutrophils isolated from tumor-bearing mice expressed higher levels of Fas/FasL, which may be involved in T cells apoptosis. However, the expression levels of Fas/FasL in TANs did not depend on the CXCL1 expression in primary tumor cells. Which factor(s) in tumor microenvironment could enhance Fas/FasL expression in TANs needs to be further investigated. Beside Fas/FasL, TANs also expressed higher levels of MPO, and the number of MPO^+^ neutrophils was less in 3LL/shCXCL1 tumor tissues than that in 3LL/NC tumor tissues. It had been demonstrated that High MPO^+^ cell infiltration in colorectal cancer was an independent favorable prognostic factor [48] and MPO inhibitor reduced lung carcinoma growth during the early stages of tumor progression [49], suggesting that MPO^+^ neutrophil involved promoting lung cancer growth. Additionally, in our unpublished studies we demonstrated that TANs expressed higher levels of PDL1, which exhibited immunosuppressive function via inhibiting CD4^+^ T and CD8^+^ T cell proliferation, which maybe contribute to TAN-mediated suppression of T cells proliferation. In conclusion, 3LL tumor-derived CXCL1 contributes to TANs infiltration in lung cancer which promotes tumor growth.

## Abbreviations

MPO: Myeloperoxidase
TANs: Tumor-associated neutrophils
MDSCs: Myeloid-derived suppressive cells
mDC: Mature dendritic cells
HCC: Hepatocellular carcinoma
IF: Immunofluorescence
IHC: Immunohistochemistry.
