# Corrigendum to “Tumor-Derived CXCL1 Promotes Lung Cancer Growth via Recruitment of Tumor-Associated Neutrophils”

**DOI:** 10.1155/2020/5106904

**Published:** 2020-08-12

**Authors:** Journal of Immunology Research

The article titled “Tumor-Derived CXCL1 Promotes Lung Cancer Growth via Recruitment of Tumor-Associated Neutrophils” [1] was reassessed due to concerns raised with research by Xuetao Cao [2]. The editorial board identified a figure duplication in the top two panels of Figure 4(c), which the authors explained was a mistake introduced during initial manuscript preparation and figure assembly.

The experiment was repeated by Dr. Mingyan Huang and supervised by Prof. Nan Li of the National Key Laboratory of Medical Immunology, Second Military Medical University. The corrected Figure 4(c) is shown below.

## Figures and Tables

**Figure 1:**
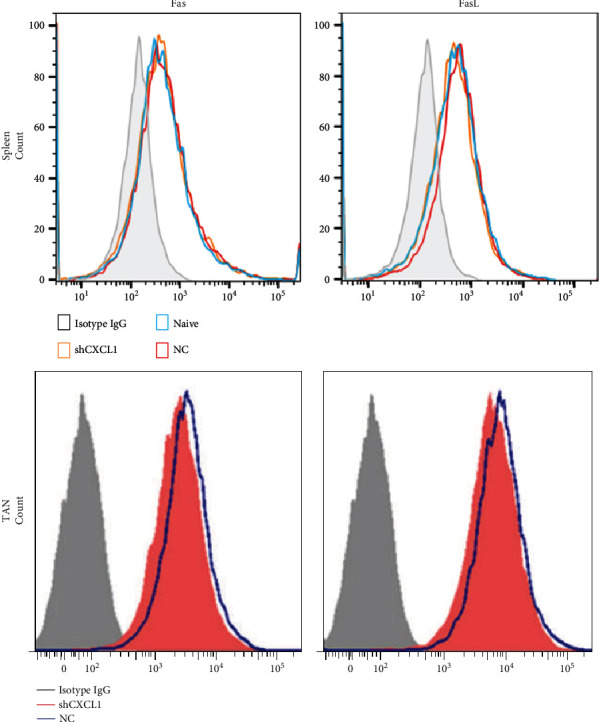
Tumor-infiltrating neutrophils in tumor inhibit T cell proliferation. (c) Fas/FasL expression on splenic CD11b^+^Ly6G^+^ neutrophils or TANs from 3LL/NC bearing mice, 3LL/shCXCL1 bearing, or naïve mice were analyzed by cytometric analysis.

## Supplementary Materials

Supplementary Materials The electronic laboratory notes for the repeated experiments are available as supplementary materials.
