# Supplementary material for: Corrigendum to “Tumor-Derived CXCL1 Promotes Lung Cancer Growth via Recruitment of Tumor-Associated Neutrophils”
Source: J Immunol Res. 2020 Aug 12;2020:5106904. doi: 10.1155/2020/5106904 (PMC7447590; doi:10.1155/2020/5106904)
Supplement: Supplementary Materials — The electronic laboratory notes for the repeated experiments are available as supplementary materials. [file 5106904.f1.pdf]

2020 年 3 月 10 日　　　　　　　　　　　　　　　　　　　　星期二

11:00 am

小鼠订购：

订购 6-8 周龄、雄性、C57BL/6J 共计 18 只；上海必凯实验动物公司

2:30pm

细胞系扩增培养：

① 小瓶中培养的三株细胞系 3LL Naïve、3LL NC(negative control)、3LL S719（silenced），细胞覆盖率均达 80-90%，可消化传代；

② 完全弃除小瓶中的培养基，每瓶加胰酶 2ml，置细胞培养箱中，取出后镜下观察看到细胞形态变圆终止消化；

③ 每瓶加 10%FCS 1640 培养基 10ml 终止消化，用 10ml 吸管将瓶内细胞转移至新的 50ml 离心管中，标记；1000rpm，离心 5min 后弃上清；

④ 细胞沉淀用新鲜 10%FCS 1640 培养基 15ml，吹打混匀后转移至新的中号培养瓶中，做好标记，于 37℃，5% CO_2 细胞培养箱中培养备用。

实验操作及记录：黄明燕

2020 年 3 月 11 日 星期三

11:30 m

1. 从上海必凯实验动物公司订购的 C57 小鼠到，放到免疫所老楼动物房屏障间备用。
2. 每组：每笼 6 只 C57 小鼠，分别用于接种 3LL、3LL NC 和 3LL S719 细胞系。

实验操作及记录：黄明燕

2020 年 3 月 13 日　　　　　　　　　　　　　　　　　　　　　　星期五

9:00 am

1. 收集培养细胞：

① 中瓶中培养 3LL、3LL NC、3LL S719 细胞株，细胞覆盖率达 80-90%，弃培养基，每瓶加胰酶 4ml 消化细胞，操作同上所述；

② 每瓶加 10% FCS 1640 培养基 20ml 终止消化，用 10ml 吸管将培养瓶内细胞全部吹下转移至新的 50ml 离心管中，做好标记，1000rpm，离心 5min 后弃去上清；

③ 细胞计数：细胞沉淀用无菌 1×PBS 吹打混匀，从中吸取 100ul 细胞悬液于新 1.5ml EP 管中，添加 900ul 无菌 PBS 混匀，从中吸取 10ul 细胞悬液加入血球计数板中，显微镜下计数；

④ 根据细胞计算调整细胞浓度为 5*10^6 /ml，备用。

10:30 am

荷瘤小鼠模型制备：

① 用 3 支 1ml 注射器，分别吸取上述准备好的细胞系 3LL、3LL NC 、3LL S719（细胞浓度均为 5*10^6 /ml）

② 按先前分好的组别于小鼠腹部皮下分别接种 3LL、3LL NC、3LL S719 细胞，每只小鼠 100ul 细胞，即每只接种细胞总数为 5*10^5 个细胞，每种细胞各接种 6 只小鼠。

③ 接种后隔天观察荷瘤小鼠情况，两周后处死小鼠，取荷瘤小鼠脾脏备用。

实验操作及记录：黄明燕

2020 年 3 月 27 日 星期五

10:00 am

荷瘤小鼠脾脏细胞制备：

① 各取 3 只 3LL、3LL NC、3LL S719 细胞荷瘤小鼠处死；

② 取出上述荷瘤小鼠脾脏置于预先放置 6ml 1×PBS 的培养皿的滤网中；

③ 研磨脾脏, 将细胞悬液转移至新的 15ml 离心管中, 做好标记, 2000rpm,4℃, 离心 5min 后弃上清；

④ 细胞沉淀添加 Tris·NH4Cl 红细胞裂解液 10ml，充分吹打混匀，静置 5min，待红细胞充分裂解; 2000rpm,4℃，离心 5min 后弃上清；

⑤ 预冷的 1×PBS 10ml 洗 3 遍；用 50ul PBS 重悬细胞；

荧光染料标记

1. 准备荧光抗体：LY6G-FITC，CD11b-PerCP-cy5.5，Fas-PE，FasL-PE；
2. 对照管设置：空白管-无标记；单标管-FITC；单标管-PerCP-cy5.5；单标管-PE
3. 实验管标记，每只小鼠脾脏所得细胞分为为两管，一管标记 LY6G-FITC，CD11b-PerCP-cy5.5 和 Fas-PE（3 标）;另一管标记 LY6G-FITC，CD11b-PerCP-cy5.5 和 FasL-PE（3 标）。
4. 每种荧光染料每管用量 0.5ul，预先将 3 种荧光抗体混合混匀，每管加入 1.5 ul，混匀后于 4℃冰箱避光孵育 30min；
5. 孵育结束后，预冷的 1×PBS 1ml 洗 3 遍，离心后用 PBS 400ul 重悬后转移至流式管中备用。

上机检测：

1. 流式细胞仪：BD LSRFoetessa；

2. 用空白管调整细胞的 FSC/SSC，单通道荧光标记管调整各荧光素对应的电压和不同荧光素之间的补偿；
3. 各荧光素电压、补偿调节好后，各管上机检测；
4. 数据保存在 BD LSRFoetessa^{TM} 流式联机电脑名为“307hmy”文件夹的 fas 子文件夹中。

实验操作及记录：黄明燕

2020 年 3 月 28 日　　　　　　　　　　　　　　　　　　　　　星期六

流式数据分析：

1. 软件分析：FlowJo v10.6.1
2. 从细胞群中选出 LY6G^{+}CD11b^{+}双阳性的细胞群，即为所要分析的中性粒细胞群。
3. 对圈选出的 LY6G^{+}CD11b^{+}双阳性细胞群显示其 PE 荧光表达，并选择峰图（Histogram）模式。
4. 将 3LL、3LL NC、3LL S719 三个细胞的 Fas-PE 和 FasL-PE 峰图分别叠加，并以无标记空白管做阴性对照，即得到实验结果图。

实验分析及记录：黄明燕

Repeated results with raw data

#1

Fas

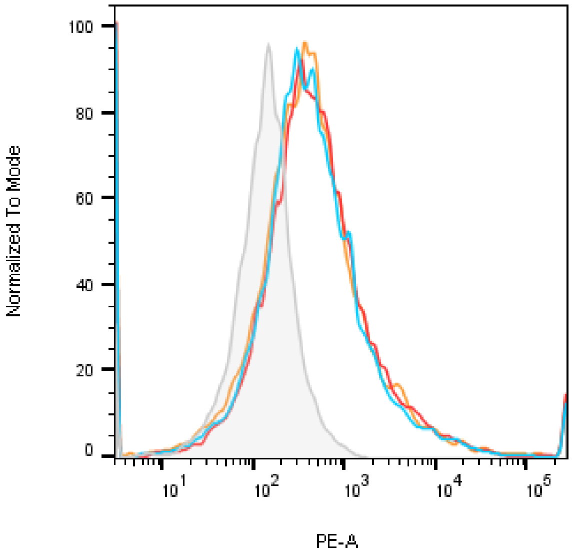

	Sample Name	Count	Mean : PE-A
	20200327 spleen_nc -_006.fcs	9745	169
	20200327 spleen_3ll fas2_011.fcs	10678	3847
	20200327 spleen_nc fas2_012.fcs	10600	4232
	20200327 spleen_719 fas2_013.fcs	9815	3979

FasL

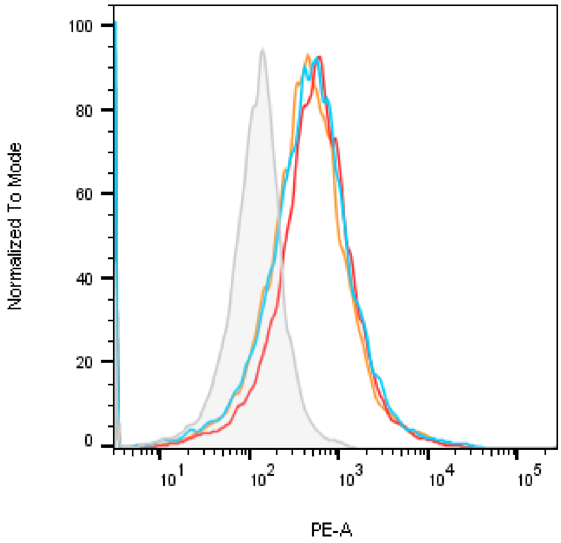

	Sample Name	Count	Mean : PE-A
	20200327 spleen_719 pe_025.fcs	10433	146
	20200327 spleen_3ll fasl-4_040.fcs	10264	993
	20200327 spleen_nc fasl-4_041.fcs	10171	1057
	20200327 spleen_719 fasl-4_042.fcs	10243	880

#2

Fas

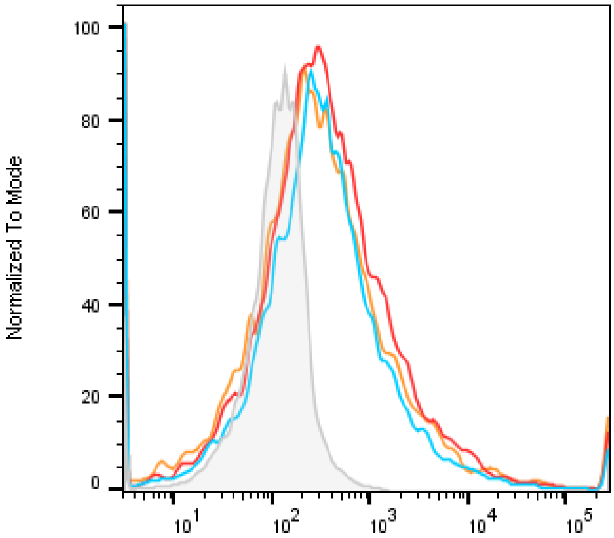

	Sample Name	Count	Mean : PE-A
	20200327 spleen_3ll -_005.fcs	11040	136
	20200327 spleen_3ll fas-1_008.fcs	10983	2891
	20200327 spleen_nc fas-1_009.fcs	11308	3263
	20200327 spleen_719 fas-1_010.fcs	10193	2699

FasL

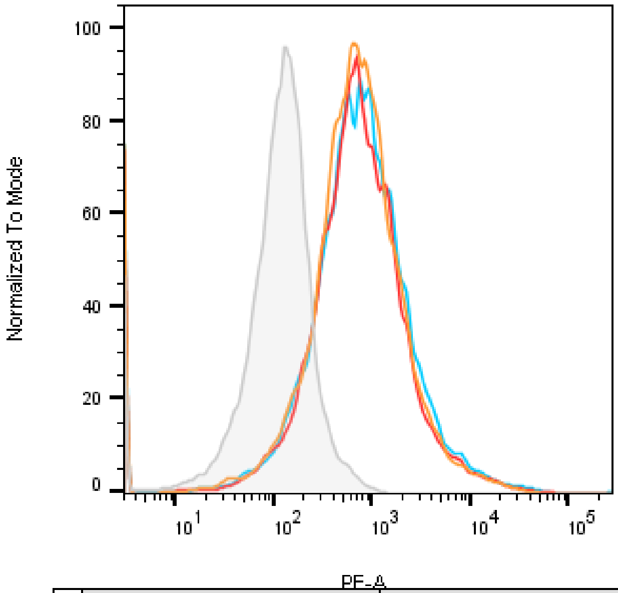

	Sample Name	Count	Mean : PE-A
	20200327 spleen_3ll pe_023.fcs	11615	144
	20200327 spleen_3ll fasl-3_037.fcs	9562	1570
	20200327 spleen_nc fasl-3_038.fcs	9597	1636
	20200327 spleen_719 fasl-3_039.fcs	9596	1718

#3

Fas

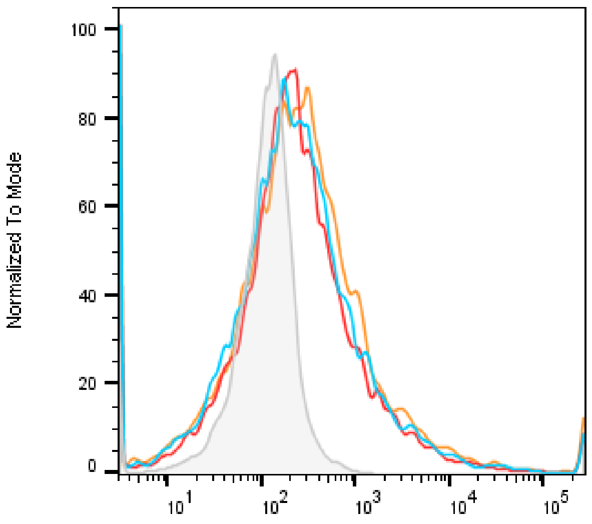

	Sample Name	Count	Mean : PE-A
	20200327 spleen_719 -_007.fcs	10436	135
	20200327 spleen_3ll fas-3_014.fcs	11188	2323
	20200327 spleen_nc fas3_015.fcs	10838	2687
	20200327 spleen_719 fas-3_016.fcs	10591	2569

FasL

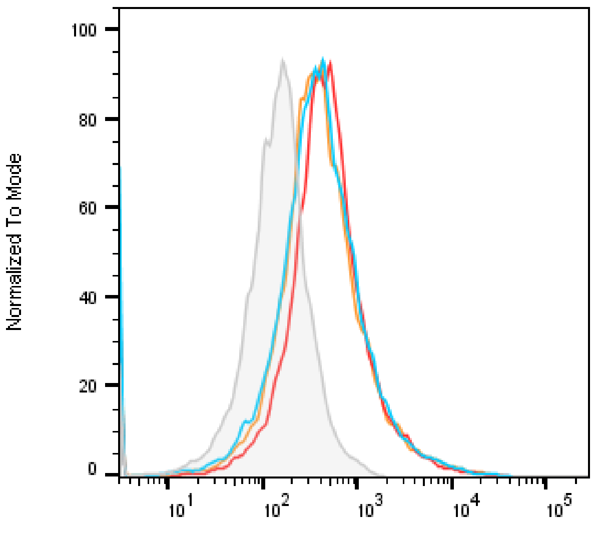

	Sample Name	Count	Mean : PE-A
	20200327 spleen_nc pe_024.fcs	9542	183
	20200327 spleen_3ll fasl_017.fcs	11314	900
	20200327 spleen_nc fasl_021.fcs	10949	946
	20200327 spleen_719 fasl_022.fcs	11066	855
